# Cigarette Smoking and Cognitive Function in Chinese Male Schizophrenia: A Case-Control study

**DOI:** 10.1371/journal.pone.0036563

**Published:** 2012-05-03

**Authors:** Xiang Yang Zhang, Da Chun Chen, Mei Hong Xiu, Colin N. Haile, Hongqiang Sun, Lin Lu, Therese A. Kosten, Thomas R. Kosten

**Affiliations:** 1 Institute of Psychology, Chinese Academy of Sciences, Beijing, People's Republic of China; 2 Menninger Department of Psychiatry and Behavioral Sciences, Baylor College of Medicine, Houston, Texas, United States of America; 3 Beijing HuiLongGuan Hospital, Peking University, Beijing, People's Republic of China; 4 The National Institute on Drug Dependence, Peking University, Beijing, China; Centre national de la recherche scientifique, France

## Abstract

Schizophrenic patients have higher smoking rates than the general population. Studies show that smoking may be a form of self-medication in an attempt to alleviate cognitive deficits in schizophrenic patients of European background. This study examined the relationships between smoking and cognitive deficits in Chinese schizophrenic patients, which have previously received little systemic study. We recruited 580 male chronic patients meeting DSM-IV criteria for schizophrenia and 175 male control subjects who were matched on age and education. The subjects completed a detailed cigarette smoking questionnaire, the Fagerstrom Test for Nicotine Dependence (FTND), and the Repeatable Battery for the Assessment of Neuropsychological Status (RBANS). Patients also were rated on the Positive and Negative Symptom Scale (PANSS), the Simpson and Angus Extrapyramidal Symptom Rating Scale (SAES), and the Abnormal Involuntary Movement Scale (AIMS). All five RBANS subscales except for the Visuospatial/Constructional index showed significantly lower cognitive performance for schizophrenics than normal controls. The schizophrenic smokers scored lower than the schizophrenic non-smokers on the RBANS total score and the Visuospatial/Constructional and Immediate Memory indices. Similarly, the control smokers scored lower than the control non-smokers on the RBANS total score and the Immediate Memory index . Also, the schizophrenic smokers consistently performed the poorest on the cognitive domains of the RBANS. Among the schizophrenic patients, smokers displayed significantly fewer negative symptoms than non-smokers. Using multivariate regression analysis the following variables were independently associated with the RBANS total score: years of education, PANSS negative symptom score, age at schizophrenia onset, and number of hospitalizations. Our results show that smoking is associated with significant cognitive impairment in both schizophrenic patients and normal controls, but the smokers with schizophrenia had a reduced level of negative symptoms, suggesting that the benefits of smoking for those with schizophrenia may be limited to certain aspects of a given clinical phenotype.

## Introduction

Schizophrenia is a severe psychiatric disorder characterized by multi-faceted deficits in neurocognitive function, including learning, memory, attention, executive functioning and cognitive processing speed [1]–[4]. Cognitive impairment is an enduring feature of schizophrenia [5] which is often present at illness onset [6], [7] and persists regardless of a change in patients' symptom state [8]. Cognitive impairments are major impediments to social rehabilitation and predict poor clinical outcome in patients with schizophrenia [2]. However, lack of effective treatment by currently available medications for cognitive symptoms remains a major unmet need in the management of schizophrenia. Hence, cognition has become a prime treatment target in schizophrenia [9].

Schizophrenia has a higher rate of cigarette smoking than patients with other psychiatric diagnoses and the general population [10]–[14]. Furthermore, smokers with schizophrenia may also have higher daily cigarette consumption, favor stronger cigarettes and extract more nicotine from their cigarettes [15], [16] than normal smokers. These observations remain true across cultures and countries and when controlling for possible confounds, such as marital and socio-economic status, alcohol use, antipsychotic use, or institutionalism [12], [17]. Some studies indicate that cigarette smoking or nicotine may ameliorate some of the cognitive deficits in schizophrenic patients [18]–[23] or may normalize deficits in auditory sensory gating in both schizophrenia and their first-degree relatives [24]–[26], suggesting that cigarette smoking or nicotine serves as a form of self-medication [27]–[30]. This observation also suggests that a dysfunction in nicotinic acetylcholine receptor signaling may be involved in the etiology of cognitive deficits in schizophrenia [31], [32].

Although evidence suggests that smoking may ameliorate a number of cognitive deficits associated with schizophrenia, the association between smoking and cognitive deficits has been under-examined among Chinese patients. To date, there has been no published study reporting this association. This is surprising since 67% and 56% of males and 7% and 6% of females were ever-smokers or current smokers respectively [33]. In addition, gender is a major determinant of smoking behaviors in all countries [12], although the gap between smoking rates in men and women, both with and without schizophrenia, has lessened considerably in North American and European countries [17]. However, in China, female smoking is extraordinarily rare in both general population (male/female: 67.1% vs 7.1%) [33], and in patients with schizophrenia (male/female: 81% vs 5%) [14]. Thus, we focused on male subjects in this study. The purpose of this study therefore was to determine if smoking was associated with improved cognitive functioning in a large Han Chinese schizophrenic inpatient population.

## Methods

### Ethics Statement

After a complete description of the study, all subjects gave their written informed consent to participate in the study. The protocol was approved by the Institutional Review Board (IRB), Beijing HuiLongGuan hospital.

To test whether the participants had the capacity to consent a psychiatrist evaluated them. The psychiatrists explained the research procedure to the subject and ensured that the research participants understood what they were being asked to do in the study through specific questions about research processes and this specific interview study. The explanation was tailored to maximize the understanding of the subject by using language appropriate to the subject's level of comprehension, and emotional readiness. If he/she was willing to assent to participate in research but was not able to understand the complexity of research processes, the researcher described presenting the parents or guardians and the subject with the same information at the same time. The parents or guardians then helped to explain the study procedures to the subject, gauging the subject's interest, and maximizing her/his understanding. In this situation, the subject's parents or guardians gave their written consent on the behalf of the subject.

### Subjects

Five hundred and eighty male schizophrenic inpatients were recruited from Beijing Hui-Long-Guan hospital, a Beijing-city-owned psychiatric hospital, and HeBei Province Veteran Psychiatric Hospital in BaoDing city, which is about 50 miles away from Beijing. All patients met the following inclusion criteria: 1) age 25–75 years, Han Chinese; 2) confirmed DSM-IV diagnosis of schizophrenia; 4) with at least 5 years of illness; 5) had been receiving stable doses of oral antipsychotic drugs for at least 12 months before entry into the study. All patients were of the chronic type, with a mean illness course of 24.7±7.6 years and with a mean duration of 7.6±6.8 years on current antipsychotic treatment. Patients were hospitalized for an average of 9.1±7.2 years. Since admission, all patients received dietetically balanced hospital meals, which were occasionally supplemented by gifts (usually fruit); patients had the opportunity for about an hour of physical exercise every day. Since these patients were hospitalized, there were restrictions on their smoking, with a fixed schedule for smoking: three or four times each day, and 30 minutes each time. During the smoking period, a patient could smoke as many cigarettes as he/she liked. The patients or their family members had to purchase the cigarettes, with occasional supplemented supplies from their friends or employers, but at very low prices for most cigarette brands. Thus, smoking was not economically limited, and for the assessment period of these baseline smoking behaviors no patients were engaged in any behavior reinforcement schedules using cigarettes. The patients in the present study could be considered a representative sample of institutionalized chronic patients with schizophrenia in China.

Age- and education-matched control subjects (n = 175) were recruited from the local community in Beijing. Current mental status and personal or family history of any mental disorder was assessed by unstructured interviews. None of the healthy control subjects presented a personal or family history of psychiatric disorder. All subjects were Han Chinese recruited at the same period from the Beijing area. Demographic data for patients and normal controls are summarized in Table 1.

**Table 1:** Demographics of male patients and male control subjects.

Category	Schizophrenia N = 580	Controls N = 175
Age (years)	47.4±9.6	47.8±12.2
Education (years)	9.6±2.3	9.5±3.1
Smoking		
Current smoker	456(78.6%)	96(54.8%)
Former smoker	20(3.5%)	18(10.3%)
Never smoker	104(17.9%)	61(34.9%)

A complete medical history, physical examination and laboratory tests were obtained from patients and control subjects. All were in good physical health. Any subjects with any abnormalities whatsoever were excluded, including 5 patients and 3 healthy controls due to cardiovascular disease, 4 patients and 3 healthy controls due to cerebrovascular disease, 3 patients and 2 healthy controls due to severe infections, and 2 patients and 2 healthy controls due to cancer. Neither schizophrenic nor control subjects were currently drug or alcohol abusers or dependent.

### Measures

Each subject filled out a detailed questionnaire that recorded general information, sociodemographic characteristics, smoking behavior, and medical and psychological conditions. In addition, a cigarette smoking questionnaire was used to record smoking history and family history of smoking from the patient and verified by family members. The Chinese translation of the standardized Fagerstrom Test for Nicotine Dependence (FTND) was employed to measure the degree of nicotine dependence [34]. Additional information was collected from available medical records and collateral data (from family and/or treating clinician). Additional visits were requested for subjects with missing or ambiguous data.

The subjects were divided into different groups based on their smoking history and the FTND scores. *Never smokers* were defined as individuals who had smoked less than 100 cigarettes during their lifetime. *Former smokers* were defined as persons who had previously smoked more than one cigarette each day but had quit smoking for more than 1 year. *Current smokers* were defined as persons who smoked one cigarette or more each day and have smoked for more than 1 year. *Ever smokers* included current and former smokers. Smoking cessation rates were measured as the ratio of former smokers to the total subjects.

### Clinical Measures

The Repeatable Battery for the Assessment of Neuropsychological Status (RBANS, Form A) [35] was individually administered to measure cognitive functioning. The RBANS is comprised of 12 subtests that are used to calculate 5 age-adjusted index scores and a total score. Test indices are Immediate Memory (comprised of List Learning and Story Memory tasks); Visuospatial/Constructional (comprised of Figure Copy and Line Orientation tasks); Language (comprised of Picture Naming and Semantic Fluency tasks); Attention (comprised of Digit Span and Coding tasks); and Delayed Memory (comprised of List Recall, Story Recall, Figure Recall, and List Recognition tasks). Our group previously translated RBANS into Chinese and the clinical validity and its test-retest reliability established among controls and schizophrenic patients [36].

Each subject came in the testing room on a separate day to be introduced to our research center by a research member and a proper training session had been performed for individual to become acclimated to the testing environment and computerized tasks. In order to reduce or eliminate the withdrawal effect of smoking that may worsen cognitive functioning in individuals who smoked cigarettes, participants who smoked were allowed to smoke cigarettes prior to testing and during breaks. Breaks were taken on request at the end of each domain test of the RBANS, thus participants were not in a state of nicotine withdrawal.

Patient psychopathology was assessed using the Positive and Negative Syndrome Scale (PANSS), which was measured by four psychiatrists who had simultaneously attended a training session in the use of the PANSS before the study began. Medication side effects were assessed by the same clinical psychiatrists. Parkinsonism and akathisia were measured with the Simpson and Angus Extrapyramidal Symptom Rating Scale (SAES), and tardive dyskinesia with the Abnormal Involuntary Movement Scale (AIMS). After training, repeated assessment showed that an inter observer correlation coefficient greater than 0.8 was maintained for the PANSS, SAES, and AIMS total score.

### Statistical Analysis

Demographic and clinical variables of the smoker and non-smoker groups were compared using *t*-test, analysis of variance (ANOVA) for continuous variables and chi-squared for categorical variables. We compared RBANS scores among the four groups using analysis of variance (ANOVA). Fisher's least significant difference (LSD) test was used to perform post-hoc pair-wise between-group comparisons. When significance was found in ANOVA, the effects of the relevant variables were added to the analysis model as covariants. Effect sizes were also calculated for the two-way comparisons. Bonferroni corrections were applied to each test to adjust for multiple testing. The relationship between the RBANS and other variables, such as PANSS, years of education, age of onset, hospitalization, antipsychotic treatment, anticholinergic drugs, smoking status and nicotine dependence shown on FTND, and side effects in the patients were examined by multivariate regression analyses. In these analyses, all variables were initially entered simultaneously to determine the overall influence, and then backward stepwise procedures were employed to determine the significant associations. SPSS version 15.0 was used to do all statistical analysis. Data were presented as mean and standard deviation (mean±SD). All p values were 2 tailed with significance level set at 0.05.

## Results

Table 1 shows the demographic data of schizophrenia and normal controls. There were no significant differences in age and education between patients and normal controls (both p>0.05). There was significant difference in smoking rates between the patients and normal controls (x^2^ = 40.5, df = 2, p<0.001). Compared to normal controls, the schizophrenic patients had a higher prevalence of current smoking (x^2^ = 38.6, df = 1, p<0.0001), and lower frequency of smoking cessation (x^2^ = 13.1, df = 1, p<0.0001).

As shown in Table 2 for the schizophrenic patients alone, smokers were older (p<0.01), and had more hospitalizations. Smokers also had significantly lower levels of negative symptoms (p<0.004). After controlling for the type, the dose (chlorpromazine equivalent) and duration of treatment of antipsychotic drugs, as well as the other related variables such as age, and the number of hospitalization, the significant difference in negative symptoms between smokers and non-smokers remained (p<0.01). We did not find significant differences in any other variables between smokers and nonsmokers (all p>0.05).

**Table 2:** Characteristics of Smoking and Nonsmoking Schizophrenic Patients.

Item	Smokers N = 456	Non-smokers N = 124	t or X^2^	df	p
Age (yrs)	48.6±9.5	46.3±11.1	2.43	1,578	<0.01
Age at onset (yrs)	23.4±4.9	23.5±4.9	0.15	1,572	ns
Education (yrs)	9.8±4.8	9.2±3.6	0.79	1,569	ns
Number of hospitalizations	4.7±2.9	3.5±2.4	2.78	1,560	<0.002
Subtypes of Schizophrenia			0.32	4	Ns
Paranoid type	142(31.1%)	40(32.3%)			
Disorganized type	40 (8.8%)	10(8.1%)			
Undifferentiated type	31 (6.8%)	7(5.6%)			
Residual type	233 (51.1%)	64(51.6%)			
Catatonic type	10 (2.2%)	3(2.4%)			
Antipsychotic types			0.78	1	0.38
Typical	113(24.8%)	26(21.0%)			
Atypical	343(75.2%)	98(79.0%)			
Neuroleptic dose (chlorpromazine equivalents, mg/day)	472±458	425±264	1.25	1,568	ns
PANSS total score	61.1±16.6	63.2±17.3	−1.68	1,560	ns
P subscore	12.9±5.9	12.6±5.6	0.53	1,560	ns
N subscore	22.3±7.3	24.4±8.4	−2.97	1,560	0.004
G subscore	25.9±6.5	26.2±7.1	−0.99	1,560	ns
Parkinsonism score	1.4±2.0	2.1±2.2	−1.76	1,561	ns
AIMS total score	4.9±4.6	4.7±4.8	0.42	1,560	ns
BMI (kg/m^2^)	24.6±3.8	24.8±4.3	−0.68	1,252	ns

**Note:** PANSS = Positive and Negative Symptom Scale; P = PANSS positive symptom subscale; N = PANSS negative symptom subscale; G = PANSS general psychopathology subscale. AIMS = Abnormal Involuntary Movement Scale (AIMS). BMI = body mass index.

The following variables were significant (*P*<0.05) predictors of the RBANS total score in the schizophrenia patients: PANSS negative symptoms, years of education, PANSS total score, hospitalization times, age at schizophrenia onset, PANSS general psychopathology, antipsychotic types (atypical vs. typical drugs), anticholinergic drugs, smoking status (smokers vs. non-smoker), FTND total score and Simpson-Angus Extrapyramidal Symptoms. While age was not correlated with the total RBANS, age showed a significant negative association with immediate memory (r = −0.13, df = 1, 580. p<0.002) and attention (r = −0.11, df = 1,580, p<0.005). Using multivariate regression analysis the following variables were independently associated with the RBANS total score: years of education (t = 7.8, p = 0.0001), PANSS negative symptom score (t = −3.8, p = 0.001), age at schizophrenia onset (t = 3.0, p = 0.003), and number of hospitalizations (t = −2.1, p = 0.04). These factors together predicted 28% of the variance of the RBANS total score and were entered into the regression analyses in comparing cognitive functioning in smokers vs non-smokers.

### Cognitive Functioning in Smokers and Non-smokers: Case-Control Comparison

RBANS total and index scores of 456 smokers with schizophrenia, 124 nonsmokers with schizophrenia, 96 control smokers and 79 control nonsmokers are shown in Table 3. ANOVA revealed that diagnosis (patient vs control) differences were significant for the RBANS total scores and nearly all of its five index scores (all p<0.001) except for the Visuospatial/Constructional Index (p = 0.07). Pair-wise post hoc comparisons showed significant differences in the RBANS total score and all index scores (all p<0.05) between the schizophrenic and control non-smokers (all p<0.0001) with effect sizes ranging from 0.96 to 1.39, and between the schizophrenic and control smokers (all p<0.001) except for the Visuospatial/Constructional index (p>0.05) with effect sizes ranging from 0.36 to 1.26 (Table 4).

**Table 3:** Total and index scores on the RBANS for smokers and non-smoker in schizophrenia versus controls.

	Schizophrenia		Controls		Smoking F (p valule)	Diagnosis F (p value)	Smoking×Diagnosis F (pvalue)
	Smokers N = 456	Non-smokers N = 124	Smokers N = 96	Non-smokers N = 79			
Immediate Memory	57.2±15.1*	60.3±18.4	73.0±15.6**	78.6±17.1	6.04 (0.014)	139.8(<.001)	0.35(0.56)
Attention	69.3±17.1	71.0±18.9	87.9±15.6	92.2±20.7	2.41(0.12)	177.3(<.001)	0.06(0.81)
Language	80.7±15.4	82.5±15.7	94.2±10.0	96.8±13.4	2.18(0.14)	116.5(<.001)	0.08(0.78)
Visuospatial/Constructional	75.9±18.2**	81.2±20.1	81.0±15.6	82.1±15.8	2.84(0.09)	3.47(0.07)	1.52(0.22)
Delayed Memory	65.7±18.7	65.5±19.5	84.9±14.0	89.3±14.2	0.09(0.76)	162.0(<.001)	0.89(0.34)
Total	63.0±13.9*	66.2±16.4	79.1±11.6**	84.2±15.3	6.09(0.014)	183.9(<.001)	0.05(0.82)

**Note:** * indicates comparison between smoker and nonsmoker pairs in both schizophrenia and controls. *p<0.05; ** p<0.01.

**Table 4:** Pairwise comparisons for the RBANS index and total scores.

	Immediate Memory	Attention	Language	Visuospatial/constructional	Delayed memory	Total
*Smokers vs. non-smokers in schizophrenia*						
Effect size	0.18	NS	NS	0.28	NS	0.21
*P* value	0.057	0.35	0.22	0.004	0.98	0.029
*Smokers vs. non-smokers*						
Effect size	0.34	NS	NS	NS	NS	0.18
*P* value	0.013	0.11	0.23	0.69	0.11	0.019
*Schizophrenic Smokers vs. control Smokers*						
Effect size	1.03	1.14	1.04	0.36	1.16	1.26
*P* value	0.0001	0.000	0.000	0.011	0.0000	0.000
*Schizophrenic Non-smoker vs. control Non-smokers*						
Effect size	1.17	1.07	0.96	NS	1.39	1.13
*P* value	0.0001	0.000	0.000	0.73	0.0000	0.000

**Note:** Post hoc pairwise analyses were performed with the Scheffe' test.

ANOVA also revealed overall main effects for smoking in the RBANS total score (F = 6.09, df = 1, 751, p = 0.014), and immediate memory index (F = 6.04, df = 1, 751, p = 0.014). Smokers performed worse than nonsmokers on these domains.

Furthermore, we examined smokers and nonsmokers separately in the patient and control groups (Table 4). The schizophrenic smoker group scored lower than the schizophrenic non-smoker group on the RBANS total score (p<0.05) and the Visuospatial/Constructional index (p<0.005), and a trend toward a significant difference on the Immediate Memory index (p = 0.057).

Because of the relatively older age, chronic symptoms, long history of antipsychotic treatment and multiple hospitalizations in this sample, we also examined the impact of these covariates on cognitive performance. After controlling for age, number of hospitalizations, PANSS total score and negative symptom subscores, as well as antipsychotic drugs and anticholinergic drugs among the patients, both the overall RBANS and the Visuospatial/Constructional index remained significant. Similarly, the control smoker group scored lower than the control non-smoker group on the Immediate Memory index (p<0.05) and the RBANS total score (p<0.05) after controlling for age, which was negatively correlated with both immediate memory (r = −0.25, df = 1,175, p<0.001) and total RBANS score (r = −0.24, df = 1,175, p<0.001) among the controls.

## Discussion

To our knowledge this is the first study to compare the cognitive functioning of smokers and non-smokers in both schizophrenia patients and healthy controls within a Chinese sample. We found significantly lower cognitive scores on the RBANS and nearly all of its five subscales except for the Visuospatial/Constructional index and a total score of RBANS than normal controls. Furthermore, we found significant differences in some domains of the RBANS between smoker and non-smoker groups with and without schizophrenia. Our study also showed that the smoking schizophrenia group consistently performed the poorest on the cognitive domains of the RBANS.

Schizophrenia had significantly lower cognitive performance on nearly all of its five subscales except for the Visuospatial/Constructional index and a total score of RBANS than normal controls. The difference in the RBANS total score between schizophrenia patients and normal controls amounted to approximately 17 points and 7 points after adjusting for confounds (i.e. education and age). These results are consistent with the majority of studies assessing cognitive performance in patients with schizophrenia and in particular with a recent USA study by Dickerson [37]. However, Dickerson's study also found a significant difference on the Visuospatial/Constructional index. This difference may reflect the different demographic and clinical states of the two samples, since Dickerson included female outpatients stabilized on psychotropic medication for as little as 3 weeks. In contrast, our exclusively male inpatient sample was on stable antipsychotic medications for at least 12 months. Our patients were also older and less educated. While our controls were matched on age, their controls were substantially younger likely leading to better performance on the RBANS tests (46 vs 36 years old). The importance of these gender, age and education effects on the RBANS profiles of subtests among schizophrenia was emphasized in a recent study of this instrument [38].

Further, performance on some measures of cognitive function was inferior to that of non-smokers in both schizophrenic and control groups, which was in contrast to our original expectation. A previous study by Jockers-Scherübl et al [4] showed that regular cannabis abuse prior to the first psychotic episode improved cognition in some tests in schizophrenic patients, but deteriorated test performance in healthy controls, suggesting that regular cannabis abuse may have a different effect on cognitive function in schizophrenic patients and healthy controls. However, in our present study, smokers appear to display decreased cognitive functions in both schizophrenic patients and healthy control subject. Whether the different substance abuses may produce the differential effects on the cognitive functions of schizophrenic patients and healthy controls deserves the further investigation.

Nicotine is currently being used to treat Alzheimer's disease (AD) and Parkinson's disease (PD), and the beneficial effects of nicotine have been attributed to an up-regulation of nicotinic acetylcholine receptors (nAChRs), or possibly a protection from the neurotoxicity induced by free radicals [39], [40]. However, nicotine also can produce neuronal toxicity [41]. It has shown that chronic cigarette smoking is a potent stimuli of oxidative processes including increased free radical production and antioxidant depletion [42]. For example, nicotine has decreased glutathione (GSH) levels and increased malondialdehyde (MDA), an indicator of lipid peroxidation in Chinese hamster ovary cells [41], [42]. Homogenized rat pancreas showed a positive correlation between nicotine dose and lipid peroxidation, and this lipid peroxidation from nicotine was abolished by adding SOD and catalase [43]. Also, in human cigarette smoking has increased lipid peroxidation and impaired antioxidant systems [44]. Increased levels of serum MDA and decreased paraoxonase activities were found in healthy smokers [45]. Thus, smoking may enhance oxidative stress not only through the production of reactive oxygen radicals in smoker, but also through weakening of the antioxidant defense systems. Overall, nicotine's effects may be dose dependent, and low dose nicotine may be an antioxidant and be neuroprotective, whereas high dose nicotine may induce neurotoxicity through oxidative stress and cellular injury [46]. In addition, the other 4700 components of tobacco smoke include a variety of carcinogens as well as other toxic compounds such as carbon monoxide, heavy metals and cyanide, which are neurotoxic [47], and could account for some of the cognitive, intellectual, and behavioral impairments in the nondemented elderly [48], and in children [49]. One recent study reported that using event-related functional MRI (fMRI), prefrontal attentional network activity was significantly reduced in smokers compared to nonsmokers in young adults. Furthermore, in smokers, the history of smoking duration (years) was found to be directly related to the extent of diminished attentional network activity, suggesting that chronic nicotine abuse may be sufficient to exert long-lasting neurotoxic effects on brain function of adolescents and young adults [50]. Taken together, cognitive impairment in both schizophrenia and controls who smoke in our present study may be associated with neuronal toxicity produced by nicotine, especially at high dose.

In this study we also found that the schizophrenic smokers displayed significantly lower negative symptoms. This finding replicates earlier work in schizophrenic patients from western regions [51], but is not consistent with others [52]. We postulate that the neurobiological mechanism for our finding of lower negative symptoms in smokers than non-smokers with schizophrenia might be associated with the increased dopamine (DA) function caused by cigarette smoking. Nicotinic acetylcholine receptors have been identified on mesolimbic and nigrostriatal dopaminergic neurons [53]. In rats, acute administration of nicotine stimulates release of dopamine in the striatum and nucleus accumbens by acting on presynaptic nicotine receptors [54] and chronic nicotine treatment decreases dopamine catabolism in the dorsal striatum [55]. Since the negative symptoms of schizophrenia are associated with hypoactivity of dopaminergic systems, smoking may lessen negative symptoms by increasing dopamine in the nucleus accumbens [56]. However, this is only our speculation. The exact mechanisms for association between smoking and reduced negative symptoms deserve further investigation.

There are several factors that limit the findings of the present study. First, this is a cross-sectional study design and cannot show direct causality of smoking, whether beneficial or harmful, in patients with schizophrenia. Second, the ability to generalize our study is limited by our sample of chronically hospitalized male patients, who had more severe psychopathology and longer duration of illness than typical psychotic outpatients or first episode and drug-naïve patients with schizophrenia. Third, these patients had a wide age range including older adults aged upwards of 75 years old. The sample was in general an older one with the mean age of 48 and standard deviation of 10 years. The impact of aging on cognitive decline is clear. In the control group, age had a significant negative association with attention, immediate memory and RBANS total score; in the patient group, age was negatively associated with immediate memory and attention. Also, these patients had a long history of almost 20 years of treatment with antipsychotics, which may have influenced the effects of smoking to some degree. However, after statistical control for these effects of age and antipsychotic treatment, the association between smoking and cognition remained significant. Nevertheless, analysis in a younger and a less chronic patient population would be worthwhile. Fourth, our sample was limited to only males and can not be applied to females. Fifth, no drug screening was performed among the subjects in the study which would have been prudent since use of non-prescription drugs definitely has an impact on performance on cognitive tasks [57]. Sixth, the participants who smoked were allowed to smoke cigarettes during breaks in the cognitive testing in order to avoid the effect of nicotine withdrawal; however, a formal test of nicotine withdrawal was not included. We also did not record any data about the numbers of cigarettes smoked during breaks for each group. Future studies might look at whether the schizophrenics seek out more cigarettes per session than controls.

In conclusion, our results show that smoking is associated with significant cognitive impairment in both schizophrenic patients and controls. However, schizophrenic patients who smoked displayed significantly fewer negative symptoms. These results suggest that the benefits of smoking for those with schizophrenia may be limited to certain aspects of a given clinical phenotype such as negative symptoms, but not to cognitive functioning. Further studies in first-episode and drug naïve patients with schizophrenia would help clarify the interrelationship between clinical symptoms, cognitive functioning and smoking.
